# Characteristics of dynamic cerebral autoregulation in cerebral small vessel disease: Diffuse and sustained

**DOI:** 10.1038/srep15269

**Published:** 2015-10-15

**Authors:** Zhen-Ni Guo, Yingqi Xing, Shuang Wang, Hongyin Ma, Jia Liu, Yi Yang

**Affiliations:** 1 Neuroscience Center, Department of Neurology, the First Hospital of Jilin University, Chang Chun, China; 2 Center for Neurovascular ultrasound, the First Hospital of Jilin University, Chang Chun, China; 3 Shenzhen Institutes of Advanced Technology, Chinese Academy of Sciences, Xueyuan Avenue, Shenzhen University Town, Shenzhen, China

## Abstract

Cerebral small vessel disease is a major cause of stroke and vascular dementia; however, the pathogenesis is largely unclear. In this study, we investigated the characteristics of the impairment of dynamic cerebral autoregulation (dCA) in lacunar infarction patients. Seventy-one lacunar infarction patients were enrolled in the study, including 46 unilateral middle cerebral artery (MCA) territory stroke patients and 25 unilateral posterior cerebral artery (PCA) territory stroke patients. Each group of patients was randomly divided into two subgroups. Group 1 underwent dCA assessments in the bilateral MCAs, and Group 2 underwent dCA assessments in the bilateral PCAs. All patients were followed up for 6 months. Transfer function analysis was applied to derive the autoregulatory parameters of gain and phase difference. In the unilateral MCA territory stroke patients, impairments of dCA were observed in both the MCAs and PCAs, and the same results were observed in the unilateral PCA territory stroke patients. These impairments remained unchanged during the 6-month follow-up. In lacunar infarction, which is most prevalent type of cerebral small vessel disease, though patients with unilateral MCA territory/PCA territory stroke, the impairments of dCA were global and sustained. This finding suggests that the physiological changes associated with lacunar infarction were diffuse.

The term “cerebral small vessel disease” refers to the syndrome of clinical, cognitive, neuroimaging, and neuropathological findings that are thought to arise from diseases that affect the perforating cerebral arterioles, capillaries and venules in the brain^1^,^2^. Cerebral small vessel disease is a major cause of stroke, age-related cognitive decline, and vascular dementia. The pathogenesis of cerebral small vessel disease, which might involve arteriolosclerosis, is largely unclear^3^,^4^,^5^.

Dynamic cerebral autoregulation (dCA) is an indicator of vascular function. Impairments of dCA are associated with many common diseases, such as stroke^6^, Alzheimer’s disease^7^ and right-to-left shunts^8^. In our previous study, we found that in lacunar infarction, which is one of the characteristics of cerebral small vessel disease, dCA is evidently impaired. Other studies have also reported that static cerebral autoregulation/dCA is impaired in lacunar infarction patients, but these studies have only proven that the impairments of CA in lacunar infarction occur in one or two sides^6^,^9^,^10^. Risk factors, such as hypertension, diabetes, etc^5^,^11^., can cause diffuse damage to the cerebral small vessels and affect the function of the vessel walls; thus, impairments of dCA might have global rather that only local effects. In the present study, we sought to confirm this hypothesis by assessing dCA in the bilateral middle cerebral arteries and posterior cerebral arteries of unilateral middle cerebral artery and posterior cerebral artery territory lacunar infarction patients (Fig. 1). Observations of impairments of dCA in the primary intracranial arteries would confirm global impairment.

## Results

### Demographic information

In total, 46 unilateral middle cerebral artery territory stroke patients (54.39 ± 9.48 years; 33 males and 13 females) were enrolled in the study. Twenty-five patients underwent dCA assessments in the bilateral middle cerebral arteries, and the remaining 21 patients underwent dCA assessments in the bilateral posterior cerebral arteries. Twenty-five unilateral posterior cerebral artery territory stroke patients (52.96 ± 10.14 years; 17 males and eight females) were enrolled; 12 of these patients underwent dCA assessments in the bilateral middle cerebral arteries, and 13 patients underwent dCA assessments in the bilateral posterior cerebral arteries.

In the middle cerebral artery stroke group, 31 patients (67.4%) had basal ganglia region infarction, 10 (21.7%) had temporal lobe infarction, and 5 (10.9%) had frontal lobe infarction. In the posterior cerebral artery stroke group, 12 patients (48.0%) had thalamus infarction, 9 (36.0%) had temporal lobe infarction, and 4 (16.0%) had occipital lobe infarction. Thirty medically and psychiatrically healthy volunteers (50.60 ± 9.84 years; 20 males and 10 females) served as controls; 20 of these volunteers underwent dCA assessments in the bilateral middle cerebral arteries, and 10 underwent dCA assessments in the bilateral posterior cerebral arteries. There was no difference of baseline among these groups. The baseline characteristics are presented in Table 1.

### Dynamic cerebral autoregulation

In the middle cerebral artery control group of healthy volunteers, the phase differences (PDs; 0.06–0.12) between the arterial blood pressure and CBFV were 63.52 ± 21.75 degrees in the left hemisphere and 66.40 ± 21.17 degrees in the right hemisphere, and these values were not significantly different (t = −0.87, *p *> 0.1). The overall PD in this group was 64.96 ± 21.24 degrees. The gains were 0.69 ± 0.26 in the left hemisphere and 0.67 ± 0.34 in the right hemisphere, and these values were not significantly different (t = 0.40, *p *> 0.1). The overall gain was 0.68 ± 0.30. In the posterior cerebral artery control group, the PDs were 62.62 ± 13.69 degrees in the left hemisphere and 60.69 ± 12.96 degrees in the right hemisphere, and these values were not significantly different (t = 0.47, *p *> 0.1). The overall PD in this group was 61.66 ± 13.01 degrees. The gains were 0.68 ± 0.18 in the left hemisphere and 0.58 ± 0.18 in the right hemisphere, and these values were not significantly different (t = 1.24, *p *> 0.1). The overall gain was 0.63 ± 0.18.

In the middle cerebral artery territory stroke group, the PDs in the middle cerebral arteries of the affected and unaffected hemispheres were similar (36.10 ± 19.65 vs. 34.20 ± 21.09, respectively, t = 0.877, *p *> 0.1). The PDs in the affected and unaffected posterior cerebral arteries were also similar (36.75 ± 22.51 vs. 37.19 ± 19.59, respectively t = −0.156, *p *> 0.1). The PDs in all arteries on both sides were lower in the infarction patients than in the corresponding vessels of the healthy group (all *p* < 0.001; Table 2, Figs 2 and 3).The gains in the affected and unaffected hemispheres were similar in the middle cerebral arteries (0.61 ± 0.28 vs. 0.58 ± 0.26, t = 0.822, *p *> 0.1) and in the posterior cerebral arteries (0.43 ± 0.20 vs. 0.49 ± 0.22, t = −1.544, *p *> 0.1). Bilaterally, the gains in the posterior cerebral arteries were lower in the infarction patients than in the corresponding vessels of the healthy group (*p *< 0.05; Table 2, Figs 2 and 3).

In the posterior cerebral artery territory stroke group, the PD results were similar to those of the middle cerebral artery territory stroke group. The PDs were similar in the affected and unaffected hemispheres in the middle cerebral arteries and the posterior cerebral arteries, and the PDs of all arteries in both hemispheres were lower in the infarction patients than in the corresponding vessels in the healthy group. There were no differences in gain between the groups or sides (Table 2, Figs 2 and 3).

### Follow-Up

All patients were followed up for 6 months. No patient died during the follow-up period. A total of seven patients (9.86%; 5 in the middle cerebral artery territory stroke group and two in the posterior cerebral artery territory stroke group) were lost to follow-up. The results were similar to those obtained at the onset of stroke (paired t-tests following the deletion of the missing values, all *p *< 0.05, Table 3).

## Discussion

In the present study, we found that in unilateral middle cerebral artery territory stroke patients, dCA impairments were observed in both of the middle cerebral arteries and posterior cerebral arteries, and the same results were observed in the unilateral posterior cerebral artery territory stroke patients. These impairments remained unchanged at the 6-month follow-up. These results indicated that the dCA impairments in lacunar infarction were global and sustained. These findings agree with our hypothesis.

Lacunar infarctions account for 20–30% of all stroke subtypes, have an incidence of approximately 33 per 100,000 persons/years^12^,^13^ and are the most prevalent type of cerebral small vessel disease^14^. Static cerebral autoregulation/dCA has been reported to be impaired in lacunar infarction patients. Molina *et al.* found that chemical vasomotor cerebral autoregulation is impaired in lacunar infarction^9^, and de Leeuw *et al.* found that static cerebral autoregulation is impaired in both hemispheresin lacunar infarction patients^15^. Immink *et al.* found that dCA is bilaterally affected in the middle cerebral arteries of unilateral lacunar infarction patients, and these findings are consistent with those of our previous study^6^. These authors speculated that this type of impairment of dCA results from cerebral small vessel atherosclerosis^5^,^11^. The previous studies are meaningful, but these studies only proved that the impairments of CA in lacunar infarction occur on one or both sides. In the present study, we conducted deeper research; we chose patients with unilateral middle cerebral artery or posterior cerebral artery territory strokes and assessed the dCAs in the bilateral middle cerebral arteries and posterior cerebral arteries. The results have clarified the global impairment of dCA that occur in these patients. Additionally, we observed dCA the impairments 6 months after stroke. The volumes of lacunar infarctions are small and unlikely to cause such extensive and permanent effects on dCA alone. Therefore, we believe that it is more likely that dCA impairment is an important risk factor that leads to acute lacunar infarction rather than being caused by acute lacunar infarction.

The pathological mechanism of this global impairment of dCA might be related to arteriolosclerosis^14^, which is characterized by the loss of smooth muscle cells from the tunica media, deposits of fibro-hyaline material, narrowing of the lumen, and thickening of the vessel wall^14^ and results in decreased elasticity and increased stiffness of the arteries^16^,^17^. Consequently, the systolic and diastolic functions of the cerebral small vessels are decreased. Therefore, dCA is impaired, which leads to the inhibition of the clearance of emboli, uncontrolled CBF, and eventually embolisms^18^,^19^. Because cerebral small vessel disease is diffuse and affects the small arteries, arterioles, capillaries and venules^1^, the impairment of dCA might global despite the infarcts areas being limited. This supposition was confirmed in the present study.

Based on our results, attention should be given to the fact that lacunar infarctions are always ignored in clinical work because they are less severe than other types of stroke in terms of large-artery atherosclerosis during the acute phase and short-term prognosis^20^,^21^ however, patients with lacunar infarctions are at higher risks of suffering from cognitive decline and dementia than patients with strokes of other types^22^,^23^ and these risks can seriously affect the quality of life. Worse, no specific treatment for the acute phase of this type of stroke has been proposed^1^. To some extent, lacunar infarctions are more dangerous than other, treatable types of stroke.

This study has some limitations. First, we only examined a single type of cerebral small vessel disease; to reduce confounding biases, other types, such as cerebral white matter lesions, deep brain hemorrhages, and cerebral microbleeds, were not included in this study. Therefore, this study is insufficiently comprehensive. Second, the high prevalence of stroke in men in China^24^ and gender-based differences in the sufficiency of the bilateral temporal bone window for insonation^25^ resulted in a greater number of men than women in this study, which might have led to a gender bias. Additionally, the sample size was small, and the drop-out rate was high. Additional studies are necessary to explore the correlation between the dCA and lacunar infarction.

In summary, we found that in lacunar infarctions (which are the most prevalent type of cerebral small vessel disease), the impairments of dCA were global and permanent. These results suggest that the pathological changes associated with lacunar infarction were diffuse.

## Methods

The methods were carried out in accordance with the approved guidelines. The study design was approved by the ethics committee of the First Norman Bethune Hospital of Jilin University. Written informed consent was obtained from all subjects. We performed a prospective study of consecutive admissions to the Department of Neurology at the First Hospital of Jilin University from October 2013 to August 2014. The patients who met the following criteria were included in this study: (1) a first-occurrence symptomatic stroke; (2) unilateral middle cerebral artery or posterior cerebral artery territory lacunar infarction (excluding the brainstem); (3) absence of carotid artery or intracranial artery stenosis or occlusion; and (4) sufficient bilateral temporal bone window for insonation of the middle cerebral artery. Patients who were unable to sufficiently cooperate with the dCA examination, those with histories of atrial fibrillation, myocardial infarction, unstable angina, diabetes mellitus, autonomic disturbance, and those who were taking any medication known to affect the cardiovascular or autonomic nervous system at the time of the study were excluded. Extracranial and intracranial artery stenosis and occlusion were diagnosed by transcranial Doppler (TCD, MultiDop X2, DWL, Sipplingen, Germany), carotid ultrasound (IU22, Phillips, Andover, Massachusetts, USA) and magnetic resonance angiography. Each patient was diagnosed with a lacunar infarction and classified by two neurologists according to the clinical symptoms and magnetic resonance imaging results. Atrial fibrillation, myocardial infarction and/or unstable angina were excluded by the cardiologists. Thirty age- and sex-matched healthy volunteers were recruited as normal controls.

**Figure 1:**
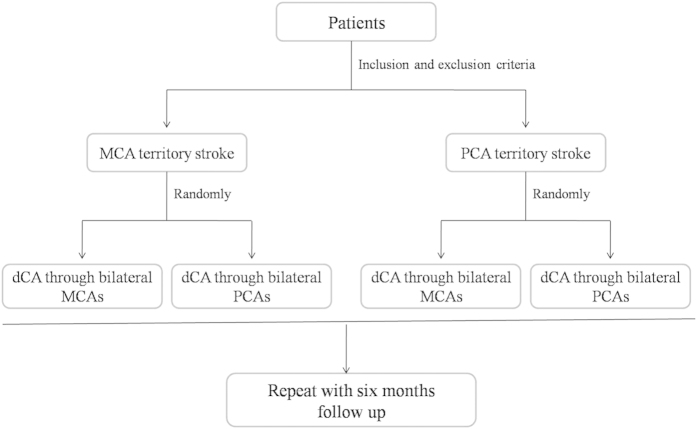
Study design. The middle cerebral artery territory stroke patients and posterior cerebral artery territory stroke patients were randomly divided into two groups. Group 1 underwent dCA assessments of the bilateral middle cerebral arteries, and Group 2 underwent dCA assessments of the bilateral posterior cerebral arteries. All of the patients were followed up at 6 months and with repeated dCA examinations.

**Figure 2:**
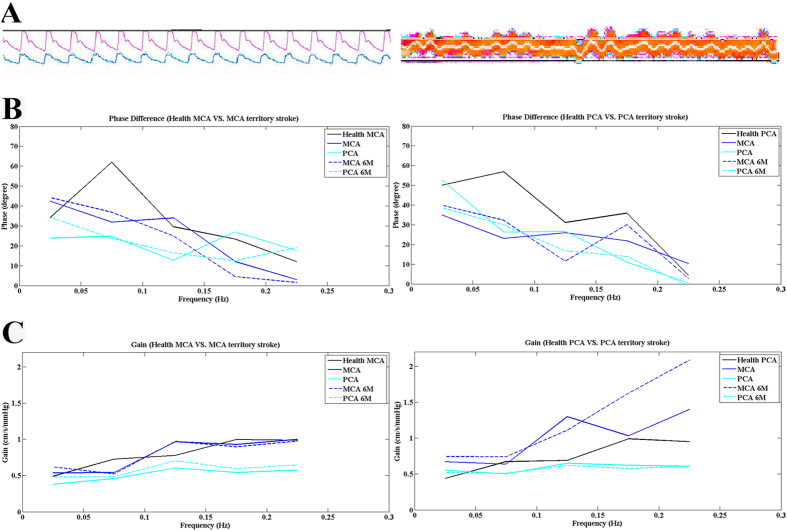
The autoregulatory parameters derived from the transfer function are plotted. (**A**) Continuous cerebral blood flow and arterial blood pressure images in the examination of dynamic cerebral autoregulation. The left is the continuous arterial blood pressure (red) and continuous cerebral blood flow (green and blue); the right is real-time continuous arterial blood pressure. (**B**) Phase differences in the middle cerebral artery and posterior cerebral artery territory stroke groups and the healthy group. In both stroke groups, the PDs of all of the arteries on both sides were lower than those of the corresponding vessels of the healthy group (within 0.06–0.12 Hz). The gains of each group (within 0.06–0.12 Hz) are presented in (**C**).

**Figure 3:**
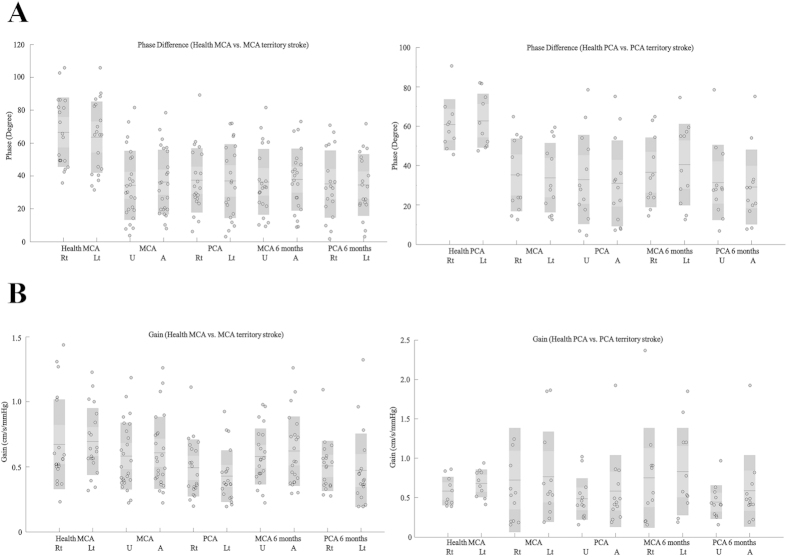
Statistical distributions of the autoregulatory parameters according the categories described above. (**A**) In the middle cerebral artery and posterior cerebral artery territory stroke groups, the PDs of all of the arteries on both sides were lower than those of the corresponding vessels in the healthy group. The differences in gain for each group are presented in (**B**).

The middle cerebral artery territory stroke patients and the posterior cerebral artery territory stroke patients were each randomly divided into two groups. Group 1 underwent dCA assessments in the bilateral middle cerebral arteries, and Group 2 underwent dCA assessments in the bilateral posterior cerebral arteries. All patients received the first dCA assessment within 48 h after onset, and the second dCA assessment at 6 months after onset. Twenty healthy volunteers underwent dCA assessments in the bilateral middle cerebral arteries, and 10 underwent dCA assessments in the bilateral posterior cerebral arteries (Fig. 1).

### Study Protocol

The subjects avoided alcohol, caffeine and nicotine for at least 12 hours before the dCA examinations, which were performed in a quiet, dedicated research laboratory with a controlled temperature of 20–24 °C in which external stimuli were minimized. The subjects were asked to adopt a relaxed supine position for 10 minutes, and the baseline blood pressure was\then measured at the brachial artery (automatic blood pressure monitor, Omron 711).Continuous cerebral blood flow velocity (CBFV) and arterial blood pressure were recorded simultaneously from each subject in the supine position for 10 minutes. The recorded data were then used to assess cerebral autoregulation^6^.

The continuous arterial blood pressure was measured non-invasively using a servo-controlled plethysmograph (Finometer Pro, the Netherlands) at the middle finger. Endtidal CO2 was monitored using a capnograph attached to a nasal cannula^6^. TCD was used to measure CBFV simultaneously in the bilateral middle cerebral arteries at a depth of 45–60 mm or simultaneously in the bilateral posterior cerebral arteries at a depth of 60–70 mm. The probes were fixed with a customized head frame^6^.

### Data Analysis

The recorded data were processed with a personal computer using MATLAB (commercially available data processing software). Beat-to-beat alignment of the data was achieved using a cross-correlation function to remove the possible time lags. A 3rd-order Butterworth low-pass filter (cutoff at 0.5 Hz) was then applied as an anti-aliasing filter before down-sampling the data to 1 Hz. The dCA was evaluated using transfer function analysis^26^. The transfer function between the arterial blood pressure and CBFV was calculated as the quotient of the cross-spectrum of the two signals and the autospectrum of the arterial blood pressure in the frequency domain. Impulse and frequency responses were derived from the transfer function analysis. In the frequency domain, we estimated the phase response, gain, and coherence function within 0.06–0.12 Hz to evaluate cerebral autoregulation; derived parameters are considered to be the most relevant to this form of hemodynamics^27^. We only used the autoregulatory parameters for the subsequent statistical analysis if the coherence within 0.06–0.12 Hz was >0.5. The patients with dCA signals of insufficient quality were excluded.

### Statistical Analysis

The Statistical Package for the Social Sciences version 17.0 (SPSS, IBM, West Grove, PA, USA) was used to analyze all data. The measurement data are expressed as the mean ± the SD, and the count data are expressed as the rate (percentage). Student’s *t*-tests were used to examine the measurement data. Chi-square and Fisher’s exact tests were used to examine the count data. The level of significance was set at *p *< 0.05.

## Additional Information

**How to cite this article**: Guo, Z.-N. *et al.* Characteristics of dynamic cerebral autoregulation in cerebral small vessel disease: Diffuse and sustained. *Sci. Rep.* **5**, 15269; doi: 10.1038/srep15269 (2015).

## Figures and Tables

**Table 1:** Baseline characteristics.

	**MCA territory stroke (n = 46)**	**PCA territory stroke (n = 25)**	**Controls (n = 30)**
Male	33 (71.7%)	17 (68%)	20 (66.7%)
Age (years)	54.39 ± 9.48	52.96 ± 10.14	53.70 ± 10.61
Mean blood pressure (mmHg)	100.41 ± 17.78	97.60 ± 13.94	89.93 ± 9.65
Heart rate	71.33 ± 8.49	70.52 ± 7.53	73.20 ± 7.08
Endtidal CO2 (mm Hg)	37.26±2.47	38.19 ± 2.53	36.11 ± 2.87
NIHSS score	3.74 ± 1.90	3.88 ± 1.69	
Hyperlipidemia	19 (41.3%)	11 (44.0%)	8 (26.7%)
Smoking	25 (54.3%)	15 (60.0%)	11 (36.7%)
Excessive drinking	9 (19.6%)	6 (24.0%)	9 (30.0%)

MCA: middle cerebral artery; PCA: posterior cerebral artery.

**Table 2:** Phase differences and gains in the patients and controls.

		**MCAs PD**	**PCAs PD**	**MCAs gain**	**PCAs gain**
MCA territory stroke	Affected hemisphere	36.10 ± 19.65*		0.61 ± 0.28	
	Unaffected hemisphere	34.20 ± 21.09*		0.58 ± 0.26	
	Left		36.75 ± 22.51*		0.43 ± 0.20†
	Right		37.19 ± 19.59*		0.49 ± 0.22†
PCA territory stroke	Affected hemisphere		30.91 ± 21.84*		0.50 ± 0.25
	Unaffected hemisphere		32.82 ± 22.70†		0.48 ± 0.26
	Left	33.71 ± 17.65*		0.76 ± 0.56	
	Right	35.16 ± 18.43*		0.72 ± 0.66	
Healthy group	Left	63.52 ± 21.75	62.62 ± 13.69	0.69 ± 0.26	0.68 ± 0.18
	Right	66.40 ± 21.17	60.69 ± 12.96	0.67 ± 0.34	0.58 ± 0.18
	Overall	64.96 ± 21.24	61.66 ± 13.01	0.68 ± 0.30	0.63 ± 0.18

MCA: middle cerebral artery; PCA: posterior cerebral artery.

^*^denotes P <0.001 compared to the corresponding vessel in the healthy group.

^†^denotes P <0.05 compared to the corresponding vessel in the healthy group.

**Table 3:** Phase differences and gains in the follow-up.

		**MCAs PD**	**PCAs PD**	**MCAs gain**	**PCAs gain**
MCA territory stroke	Affected hemisphere	37.62 ± 18.83*		0.62 ± 0.27	
	Unaffected hemisphere	36.21 ± 20.02*		0.58 ± 0.21	
	Left		34.28 ± 19.79*		0.47 ± 0.28†
	Right		34.93±20.49*		0.51 ± 0.19†
PCA territory stroke	Affected hemisphere		28.95 ± 19.06*		0.58 ± 0.45
	Unaffected hemisphere		31.33 ± 19.05*		0.44 ± 0.21†
	Left	40.40 ± 20.77†		0.83 ± 0.55	
	Right	36.47 ± 17.80*		0.75 ± 0.63	

MCA: middle cerebral artery; PCA: posterior cerebral artery.

^*^denotes P < 0.001compared to the corresponding vessel in the healthy group;

^†^denotes P < 0.05compared to the corresponding vessel in the healthy group.
